# Predicting Scores on the Mini-Mental State Examination (MMSE) from Spontaneous Speech

**DOI:** 10.3390/bs12090339

**Published:** 2022-09-16

**Authors:** Alma M. Bueno-Cayo, Minerva del Rio Carmona, Rosa Castell-Enguix, Isabel Iborra-Marmolejo, Mike Murphy, Tatiana Quarti Irigaray, José Francisco Cervera, Carmen Moret-Tatay

**Affiliations:** 1 Escuela de Doctorado, Universidad Católica de Valencia San Vicente Mártir, San Agustín 3, Esc. A, Entresuelo 1, 46002 Valencia, Spain; 2 Faculty of Psychology, Universidad Católica de Valencia San Vicente Mártir, Sede Padre Jofré, Av., Ilustración nº2, 46100 Valencia, Spain; 3 School of Applied Psychology, University College Cork, N Mall, Kilbarry Enterprise Centre, T12 YN60 Cork, Ireland; 4 Pós-Graduate Program in Psychology, Pontifícia Universidade Católica do Rio Grande do Sul, Porto Alegre 91215-330, Brazil

**Keywords:** MMSE, Mini-Mental State Examination, spontaneous speech, cognitive impairment, language

## Abstract

The aim of this study was to examine the relationship between language components, such as lexical density, length, and content in terms of “Time, Space and Action”, with MMSE scores. For this reason, a group of 33 older participants, without a diagnosis of dementia, was examined, providing information regarding recent and future events. Participants with higher MMSE scores showed higher lexical density, speech length, as well as number of tokens related to Time, Place and Action in their speech. However, these differences only reach the statistical level for lexical density when participants were divided into two groups (MCI and healthy controls). Word frequency was lower for participants with MCI but this difference was not statistically significant. Lastly, lexical density was positively correlated with MMSE scores and predicted MMSE scores. These results could be of interest at the applied level in the screening of MCI.

## 1. Introduction

According to the DSM-5 (American Psychiatric Association, 2014), different variables such as attention, executive function, learning and memory, perceptual motor skills, social recognition, and language, are variables of interest in the analysis of cognitive functioning in older adults. While the study of memory is one of the main targets in the analysis of cognitive decline in cases such as Alzheimer’s disease (AD), the literature has also documented a progressive decline in language skills [1,2,3]. Obviously, this decline seems to be more pronounced in AD than in normal ageing. However, how this decline occurs in Mild Cognitive Impairment (MCI) seems to be an issue of debate [4,5]. 

Older adults suffering from AD often show underlying lexical access problems in other processes, such as semantic and episodic memory [6,7,8]. Focusing on early symptoms in the screening of cognitive impairment through language, the literature has claimed that lexical deficits might occur, such as reduction of vocabulary size or lexical repetition [9,10], among others. In this way, measures capturing semantics and the content of words are of interest. Particularly, lexical density is a measure of semantic content words relative to the total number of words in a sentence [11] that was examined in the field. The literature has described different ways to examine lexical density in speech. These methods include picture descriptions, open-ended questions, or semi-structured interviews regarding memories, routines, or hobbies, among others [2,12,13]. In addition, other variables such as word frequency effects seem to offer mixed results. First, similar frequency effects were found for younger and older healthy adults, but the speed processing was higher for older ones [14]. In a study employing a similar task to the previous one in healthy controls versus MCI groups, no differences were found other than a slowing down for the last group [15]. One should bear in mind that this task is based on a visual word recognition task; however, there are issues underlying more ecological domains, such as spontaneous language.

A meta-analysis [16] has highlighted how fluency and naming are relevant predictors of MCI and its progression to AD. To examine the relationship between language and MCI, open questions might be of interest. Its analysis can reflect the information of interest in terms of content, such as “Time, Action and Place”, and ultimately lexical density. Most of these features can be considered as components of episodic memory, which is an integration relating to memories of a specific time and place, including re-experiencing details of the participant’s actions. Previous research has pointed out that episodic deficits observed in MCI could also affect the retrieval of coherent episodic information [8]. The literature on this type of memory in MCI has claimed a reduction in episodic memory in comparison with healthy adults [17]. This reduction was explained from an anatomical point of view, describing a loss of hippocampal volume and entorhinal cortex of the medial temporal lobes [18,19].

It is not surprising that many screening tests for cognitive impairment in older adults are based on language measures [20,21]. One of the most widely used tools is Folstein’s Mini-Mental Status Examination [22], which allows international comparisons. MMSE comprises a series of questions and the performance of some actions which can be classified into five components based on verbal questions (Orientation, Registration, Attention and Calculation, Recall, and Language) [20]. It is therefore to be expected that there is a relationship between MMSE scores and language components. Thus, the aim of this study is to examine the relationship between this screening tool and components related to speech, such as speech length, lexical density and word content related to time, place, and actions.

## 2. Materials and Methods

### 2.1. Participants

Thirty-three Spanish participants volunteered to participate in the study: 20 healthy controls and 13 individuals with MCI. A total of 66.7% were women while 33.3% were men. Patients were classified as MCI using the standard diagnostic criteria regarding MMSE [22] in its Spanish adaptation [23]. The inclusion criteria were described as follows: (i) aged between 60 and 95 years old; (ii) be a native Spanish speaker and have no hearing impairment; (ii) be able to demonstrate no substantial interference with normal daily activities as determined by clinical interview; (iv) have no dementia diagnosed. Exclusion criteria also included medical or psychiatric conditions and current self-reported mood status.

The MCI group was differentiated according to the cut-off point (24 points), also considering the level of education [24]. All participants had basic or intermediate education, except for a small group with higher education (13.33%). The control group has a mean age = 69.10 (*SD* = 7.81) while the MCI was 75.30 (*SD* = 11.43). Even if the MCI group was older, these differences were not statistically significant through Mann–Whitney U test (*p* = 0.14). The study was carried out in accordance with the Helsinki Declaration. Thus, to participate in the different studies, all participants gave informed consent (approval of the committee UCV/2020-2021/163).

### 2.2. Materials and Procedure

After a sociodemographic battery of questions, the Mini-Mental State Examination (MMSE) was employed. This is a 5–10 min screening tool to assess cognitive impairment [22]. The Spanish adaptation by Lobo et al. was employed [23]. After the previous step, the open question “what are your plans for today and what are your plans for tomorrow” was formulated and responses recorded and transcribed through WAY2AGE voice-bot based on an Azure cognitive service [20]. Before the question was asked, participants were encouraged to answer the question without a time limit. The assessment was conducted in the presence of a trained psychologist or research assistant, who also reviewed the text transcription carried out by WAY2AGE.

### 2.3. Analysis

Analyses were conducted with JASP (Version 0.12.2, Amsterdam, The Netherlands). Non-parametric approaches were employed to analyse differences across MMSE groups (control versus MCI). When data were analysed together, considering MMSE a continuous variable, a parametric approach was employed. With regards to the speech components, lexical density was calculated through the proportion of content words (nouns, verbs, adjectives, and adverbs) to the total number of words. Other components were calculated using Coh-Metrix [25]. In this way, word frequency was estimated as the average word frequency for content words. Lastly, time, place and action were calculated by counting the number of tokens related to that content in the transcribed speech. In this way, time content was analysed in terms of number of words related to adverbials of time (e.g., tomorrow), place content as nouns containing places (e.g., supermarket), and actions regarding an action verb (e.g., run).

## 3. Results

The control group showed higher lexical density scores, speech length as well as a higher number of tokens related to Time, Place and Action in their speech. However, all these differences did not reach the statistical level though the Mann–Whitney U test (*p* > 0.05) except for lexical density (*p* < 0.05; Rank Biserial correlation = 0.715; CI 95% [0.453;0.864]. Of note, word frequency was lower for the control group, but this difference was not statistically significant (*p* > 0.05). Table 1 shows the different speech components across the groups. Secondly, Pearson’s correlations were carried across variables under study, MMSE scores and age. As described in Table 2, lexical density was positively correlated with MMSE scores. Moreover, MMSE scores were strongly and negatively correlated with age.

Lastly, linear multiple regression models were carried out. In a first model, Age, lexical density, Frequency (Freq), Time, Place and Action were entered as the predictors and the outcome variables was MMSE scores: F_(6,32)_ = 3.40; MSE = 49.979; *p* < 0.05; R^2^ = 0.4. It should be noted that length was not included as a predictor in model 1, considering it was highly correlated with Time, Place and Action. In this way, a second model was carried out including length as a predictor and consequently, not including Time, Place and Action components. The second model showed the highest explained percentage of the variance: F_(4,32)_ = 6.16; MSE = 79.78; *p* < 0.01; R^2^ = 0.468. Table 3 depicts the coefficients for both models, where lexical density was statistically significant for both cases and length in model 2.

## 4. Discussion

The aim of this study was to examine the relationship between language components and MMSE scores. For this reason, spontaneous language from a group of 33 participants over 60 years old was examined. Language components in the present study involved lexical density, length, and content in terms of Time, Space and Action, measured through an open question.

The results showed a positive relationship between lexical density and MMSE scores. In addition, a negative relationship between MMSE and age was found. While lexical density is a common construct of interest in developmental psychology and childhood [26], to our knowledge, the literature seems to be limited regarding cognitive impairment [27]. Nevertheless, previous literature points out how verbal tasks are an important diagnostic criterion for both AD and MCI [28].

Lexical density is a remarkably complex variable that might reflect the complexity of communication. This pilot study suggests that this complexity would decrease with cognitive decline. However, other variables might influence lexical density (e.g., participants’ background or MCI sub-profile). One should bear in mind that MCI can be described according to the type of affection: amnestic MCI (which primarily affects memory) and non-amnestic MCI (which affects other cognitive abilities) [29]. MCI profiles were considered outside the scope of the current research due to the sample size, but future lines of research should address this issue. Nevertheless, it is considered promising that lexical density predicted MMSE scores in both models.

In relation to the length variable, a higher fluency might be expected in older adults without cognitive impairment. Length was not related to MMSE scores under a Pearson’s correlation, but it was a statistically significant predictor in regression model number two. These results are not surprising, as the literature seems to show that people with early cognitive impairments may seek alternatives or strategies to cover deficits, and mixed results are expected [30]. In this case, a compensatory strategy might be hypothesised from the differences found between lexical density and length of speech.

With regards to the word frequency variable, participants with MCI seem to use more frequent words than the control group. However, this result was inconclusive, as it did not reach the statistical level of significance. This would support previous literature on the absence of differences in this field [15]. First, other variables such as processing speed have not been considered, which might be of interest for future lines of research. Secondly, and although the educational background of the participants was relatively homogeneous, the educational level should be considered in this kind of analysis, as well as other variables such as leisure (e.g., participants’ reading time per week).

Lastly, statistically significant differences were found in the content related to Time, Space and Action across the groups. However, these variables were not predictors of MMSE scores. The relationship between content and length of speech was strong but only the length predicted MMSE scores. Once again, a compensatory strategy is hypothesised for length, which would be less sensitive to content, according to the present results. Future research should address the differences between speech length and content in the progression of cognitive impairment.

Another variable of interest is the participant’s age, which was related to MMSE but was not statistically significant in the linear multiple regression models. Although this result seems to be promising towards lexical density, one should not forget that caution is advised here due to reasons of sample size and age range. Additionally, the main limitation of the current study is related to its cross-sectional nature. Further research under longitudinal design might better reflect the evolution of cognitive impairment from these early stages or by comparing these results in spontaneous language under different set-ups, such as open questions with picture descriptions that allow response standardization. In this way, the variability between participants would be reduced.

## 5. Conclusions

After examining the relationship between spontaneous language components and MMSE scores in older adults, it can be concluded that: (i) lexical density is positively related to MMSE scores; (ii) MMSE and age were not correlated with the length or content of discourse, although length was shown to be a predictor of MMSE scores in a linear regression model; and (iii) lexical density predicted MMSE scores.

These results could be of interest at the applied level, both for the screening of MCI, as well as in future longitudinal studies for profile identification.

## Figures and Tables

**Table 1:** Speech component under study (Density = Lexical Density; Speech length = Length; Word frequency = Freq.; and number of tokens related to Time, Place and Action.

	Group	Mean	*SD*	*p*
Density	Control	0.515	0.086	<0.05
	MCI	0.400	0.069	
Length	Control	162.55	114.20	0.501
	MCI	124.38	74.13	
Freq	Control	1.369	0.389	0.298
	MCI	1.491	0.366	
Time	Control	2.700	1.657	0.353
	MCI	2.077	0.954	
Place	Control	2.050	1.276	0.265
	MCI	1.615	1.502	
Action	Control	4.850	3.328	0.372
	MCI	3.462	2.258	

**Table 2:** Pearson’s correlations across variables of interest, MMSE and age *.

Variable	Age	MMSE	Length	Density	Freq	Time	Place	Action
Age	—							
MMSE	−0.344 *	—						
Length	−0.018	0.222	—					
Density	−0.295	0.488 **	−0.306	—				
Freq	−0.132	−0.103	0.313	−0.061	—			
Time	0.127	0.243	0.773 ***	−0.083	0.254	—		
Place	−0.028	0.211	0.683 ***	−0.068	0.373 *	0.666 ***	—	
Action	−0.091	0.227	0.779 ***	−0.125	0.276	0.598 ***	0.407 *	—

* *p* < 0.05, ** *p* < 0.01, *** *p* < 0.001.

**Table 3:** Coefficients in the prediction of MMSE scores. SE = Standard Error.

Model		B	SE	β	t	*p*
1	(Intercept)	24.981	8.223		3.038	0.005
	Age	−0.120	0.077	−0.252	−1.549	0.134
	Density	21.006	7.423	0.443	2.830	0.009
	Freq	−3.070	1.962	−0.253	−1.565	0.130
	Time	0.604	0.752	0.188	0.803	0.429
	Place	0.463	0.700	0.137	0.661	0.514
	Action	0.249	0.295	0.161	0.842	0.408
2	(Intercept)	20.953	7.700		2.721	0.011
	Age	−0.097	0.069	−0.204	−1.392	0.175
	Density	26.288	7.232	0.555	3.635	0.001
	Freq	−2.927	1.778	−0.241	−1.646	0.111
	Length	0.021	0.007	0.463	3.034	0.005
